# Sex-specific clinical and neurobiological correlates of fatigue in older adults

**DOI:** 10.1007/s11357-024-01259-0

**Published:** 2024-08-12

**Authors:** Marco Toccaceli Blasi, Alba Rosa Alfano, Martina Salzillo, Simona Buscarnera, Valeria Raparelli, Matteo Cesari, Giuseppe Bruno, Marco Canevelli, for the Alzheimer’s Disease Neuroimaging Initiative

**Affiliations:** 1 Department of Human Neuroscience, “Sapienza” University, Rome, Italy; 2 Department of Internal Medicine and Medical Specialties, UOC Geriatrics, “Sapienza” University, Rome, Italy; 3 Department of Translational and Precision Medicine, “Sapienza” University of Rome, Rome, Italy; 4 Ageing and Health Unit, Department of Maternal, Newborn, Child, Adolescent Health and Ageing, World Health Organization, Geneva, Switzerland; 5 Santa Lucia Foundation IRCCS, Rome, Italy; 6 National Center for Disease Prevention and Health Promotion, Italian National Institute of Health, Rome, Italy; 7 Aging Research Center, Department of Neurobiology, Care Sciences and Society, Karolinska Institutet and Stockholm University, Stockholm, Sweden

**Keywords:** Fatigue, Aging, Sex differences, Neurodegeneration

## Abstract

Fatigue is a common and distressful symptom in older people and has been associated with adverse health outcomes. Nevertheless, its sex-specific pathophysiological underpinnings and clinical correlates have been scarcely investigated. We aimed to comprehensively explore the clinical and neurobiological determinants of fatigue in cognitively unimpaired older adults. A sex-stratified analysis was conducted to look for differences in the clinical expression of fatigue among women and men. Data on cognitively normal individuals were gathered from the Alzheimer’s Disease Neuroimaging Initiative (ADNI) 2 study. Fatigue was defined based on self-report at baseline. For each participant, information on sociodemographics, comorbidities, mood, cognitive performance, frailty, and biomarkers of brain pathology was collected. Logistic regression models, stratified by sex, were conducted to explore the factors associated with fatigue. Among the 291 participants selected, 44 subjects (15.1% of the total sample) self-reported fatigue at baseline. Subjects reporting fatigue were more likely women, had higher frailty degrees, and more severe depressive symptoms than those without fatigue. Moreover, they tended to have lower MRI hippocampus volumes. Among women, those reporting fatigue exhibited higher frailty levels, worse depression, and lower MRI hippocampus volumes relative to those without fatigue. Higher frailty degrees were also observed in men reporting vs. non-reporting fatigue. In the adjusted logistic regression model, more severe depression (OR 1.64, 95% CI 1.18–2.28; *p* < 0.01) and lower MRI hippocampus volumes (OR 0.41, 95% CI 0.19–0.90; *p* = 0.03) resulted independently associated with fatigue in women, while higher frailty degrees (OR 3.10, 95% CI 1.27–7.54 per 0.1 increase in a 39-item Frailty index; *p* = 0.01) in men. Fatigue is a complex symptom with a sex-specific pattern of clinical and neurobiological correlates. A better understanding of the underlying mechanisms of these associations is warranted to develop sex-informed approaches for personalized treatments.

**Supplementary Information:**

The online version contains supplementary material available at 10.1007/s11357-024-01259-0.

## Introduction

Fatigue is a subjective feeling of “extreme and persistent mental and/or physical tiredness, weakness, or exhaustion” [1]. It ranks among the top three reported symptoms in the general population [2] and represents one of the most common complaints among older adults [3]. Its prevalence ranges from 5 to 45% [4–9] and increases with advancing age [10], affecting women more frequently than men [11]. Fatigue can significantly impair an individual’s quality of life [12], and it has been associated with a wide range of adverse health outcomes, including reduced physical activity [13], disability [14], increased risk of hospitalization [15], and mortality [16, 17]. Recently, fatigue has also been proposed as a possible attribute and indicator of vitality [18], one of the six domains constituting the intrinsic capacity of the individual under the healthy aging framework proposed by the World Health Organization [18].

Given its subjective nature, assessing fatigue might be challenging. Despite its clinical relevance among older people, there is a lack of widely accepted operational definitions and gold-standard assessment tools, so fatigue largely remains overlooked and neglected [19]. In parallel, the pathophysiological underpinnings and clinical correlates of fatigue are still scarcely characterized. Fatigue in older people could represent a normal response to stressors or result from a wide array of somatic (e.g., heart failure, anemia, cancer); psychological (e.g., depression); or iatrogenic (e.g., chemotherapy, psychotropic drugs) pathological conditions [20]. Available evidence points to multifactorial pathogenesis, resulting from the complex interplay of multiple aging-related mechanisms (i.e., low-grade inflammation, mitochondrial dysfunction, impaired sleep, autonomic nervous system abnormalities, and poor nutritional status) [20]. Identifying a definitive causal explanation of this symptom is often difficult, especially among frail older adults suffering from multiple chronic illnesses [21]. In this regard, fatigue has been proposed as a potential sign of the age-related accumulation of deficits and vulnerabilities in maintaining homeostatic reserve (i.e., a clinical expression of the frailty syndrome) [20]. Moreover, emerging evidence suggests a potential relationship between fatigue and neurodegeneration [22]. In particular, fatigue has been associated with changes in cerebrospinal fluid (CSF) biomarkers of Alzheimer’s disease (AD) neuropathology [23], alterations of brain networks in mild cognitive impairment (MCI) [24], apolipoprotein E (ApoE) genotype [25], and amyloid deposition, functional and structural changes in the hippocampus [26–28]. Nevertheless, to date, fatigue remains scarcely investigated outside the context of specific diseases (e.g., chronic heart failure [29], cancer [30], systemic lupus erythematosus [31], rheumatoid arthritis [31], inflammatory bowel disease [32], multiple sclerosis [33], Parkinson’s disease [34].

A multidimensional, comprehensive approach is thus essential for understanding, detecting, and managing this multifaceted clinical manifestation. In this context, a sex-informed framework seems necessary considering that (i) fatigue’s prevalence is different in the two sexes, with women being more commonly affected [11], and (ii) the clinical and biological correlates of fatigue explored to date (e.g., social characteristics, genetics, comorbidities, brain pathology) are mostly unbalanced by sex [35–37]. It may thus be hypothesized that sex plays a relevant role in the pathophysiology and phenotypic expression of fatigue. Moreover, there is a growing demand from the scientific community for sex-disaggregation in health research (including aging research) to avoid sex biases and improve the generalizability of findings to practice [38–41].

In the present study, we aimed to comprehensively explore the clinical and neurobiological determinants of fatigue in cognitively unimpaired older adults, with a special focus on sociodemographics, frailty, cognition, mood, structural brain changes, and genetic traits. A sex-stratified analysis was performed to identify whether sex differences in fatigue-related clinical and neurobiological correlates exist.

## Methods

### Data sources

Data used in the preparation of this study were obtained from the Alzheimer’s Disease Neuroimaging Initiative (ADNI) database (http://adni.loni.usc.edu). The ADNI was launched in 2003 as a public–private partnership, led by Principal Investigator Michael W. Weiner, MD. The primary goal of ADNI has been to test whether serial magnetic resonance imaging (MRI), positron emission tomography (PET), other biological markers, and clinical and neuropsychological assessment can be combined to measure the progression of MCI and early AD (for up-to-date information, see http://adni.loni.usc.edu).

### Participants and procedures

Data from cognitively normal participants in the ADNI 2 study (phase 2 of the ADNI project) were considered for the present analysis.

Cognitively normal subjects were defined according to the following criteria:

1. Absence of memory complaints, beyond what one would expect for age;
2. Normal memory function, documented by scoring above education-adjusted cutoffs on the Logical Memory II subscale (Delayed Paragraph Recall, Paragraph A only) from the Wechsler Memory Scale-Revised [42];
3. Mini-Mental State Examination (MMSE) [43] scores between 24 and 30 (inclusive) (exceptions could be made for subjects with less than eight years of education at the discretion of the project director);
4. Clinical Dementia Rating [44] score of 0. Memory Box score must be 0;
5. Absence of significant impairment in cognitive functions or activities of daily living;
6. Absence of any significant neurological disease.

In the ADNI 2 study, participants between 55 and 90 years old were enrolled. The detailed eligibility and diagnostic criteria can be found in the ADNI 2 protocol (http://adni.loni.usc.edu/wp-content/uploads/2008/07/adni2-procedures-manual.pdf).

### Fatigue

At baseline visit, the presence/absence of fatigue was explored through a single question: “Do you often feel tired, fatigued, or sleepy during the daytime?”. A positive response to this question was used to identify subjects experiencing fatigue.

### Sociodemographic and clinical variables

For each participant, baseline data concerning the following domains were obtained: sociodemographics (age, self-reported sex, and education); past medical history and comorbidities; general and neurological examination; global cognitive performance (MMSE); functional independence; mood (15-item Geriatric Depression Scale [GDS-15][45]); ApoE genotype.

A modified Preclinical Alzheimer’s Cognitive Composite was considered to detect subtle cognitive changes at the neuropsychological assessment. It comprised the Alzheimer Disease Assessment Scale—Cognitive Subscale Delayed Word Recall, Logical Memory Delayed Recall, MMSE, and (log-transformed) Trail-Making Test B Time to Completion [46].

A 39-item Frailty index (FI) was computed by following a standard procedure [47] (the deficits included in the FI are listed in the Supplementary Material).

### Biomarkers of brain pathology

Data on the following biomarkers were obtained from the ADNI database: CSF concentrations of amyloid beta (Aβ_1-42_), ^181^phospho-tau (^181^P-tau), and total tau (T-tau); MRI-based measurement of the hippocampus and white matter hyperintensities volumes standardized by intracranial volume; cortical glucose metabolism at the fluorodeoxyglucose (^18^FDG) PET imaging; amyloid deposition by means of ^18^F-AV-45 (i.e., florbetapir) uptake at the PET imaging. This information was referred to the assessments carried out during both the screening and baseline visits, with a maximum time interval of 28 days. A comprehensive outline of the specific diagnostic processes, protocols, and measurements can be found in the ADNI manual (http://adni.loni.usc.edu), as well as in prior research publications [48, 49, 49, 50]. The selection of biomarkers of interest was guided by existing frameworks proposing a biological definition of Alzheimer's disease [51].

### Statistical analysis

Summary statistics were calculated for the analytic sample. Categorical variables were reported as absolute values and percentages. Continuous variables were reported as means and standard deviations or medians and interquartile ranges (IQR), as appropriate. Participants were categorized according to sex and the presence or absence of fatigue at baseline. The clinical characteristics and biomarker status of subjects complaining or not complaining of fatigue were compared, both in the overall sample and within each sex group, using the *χ*^2^ test for categorical variables and the Mann–Whitney U test for quantitative variables. Variables emerging as significantly different or trending towards statistical significance (*p*-value < 0.1) from the univariate analyses on the overall sample were then included in a logistic regression model, stratified by sex, and adjusted by age and education, with fatigue as the dichotomized dependent variable. Associations were reported as odds ratios (OR) and 95% confidence intervals (95% CI). The statistical significance was set at *p* ≤ 0.05. Statistical analyses were conducted using the Statistical Package for Social Science (SPSS) for Mac (version 27).

## Results

A total of 291 participants (median age 72.5 years, IQR 8.7; 54.0% women) were considered for the present analysis. Overall, 44 subjects (15.1% of the total sample) self-reported fatigue at baseline assessment. They were more frequently women (68.2% vs. 48.6%; *p* = 0.04); had higher frailty degrees (median FI 0.22, IQR 0.10 vs. 0.16, IQR 0.13; *p* < 0.001); and exhibited higher median GDS-15 scores (median 1.0, IQR 1.7 vs. 0.0, IQR 1.0; *p* < 0.001) relative to those without fatigue. Moreover, they tended to have lower MRI hippocampus volumes (median 4.9, IQR 0.8 vs. 5.1 IQR 0.7; *p* = 0.08). No differences were instead found in terms of cognitive functioning, ApoE ε4 carrier status, biomarkers of amyloid burden, tau pathology, brain metabolism, and severity of white matter lesions (all *p*-values > 0.05).

The sociodemographic, clinical characteristics, and biomarkers status of participants according to sex and the presence/absence of fatigue are shown in Table 1. Women reporting fatigue had higher frailty degrees (median FI 0.21, IQR 0.12 vs. 0.18, IQR 0.08; *p* < 0.001), higher median GDS-15 scores (2.0, IQR 1.0 vs. 0.0, IQR 1.0; *p* < 0.001), and lower MRI hippocampus volumes (median 4.8, IQR 0.8 vs. 5.3, IQR 0.8; *p* = 0.003) relative to women without fatigue. Higher FI scores were also observed in men reporting vs. not reporting fatigue (median FI 0.23, IQR 0.13 vs. 0.15, IQR 0.13; *p* < 0.001).

**Table 1:** Sociodemographic and clinical characteristics of participants according to sex and the presence/absence of fatigue

	Women		Men	
	Fatigue (*n* = 30)	No fatigue (*n* = 137)	Fatigue (*n* = 14)	No fatigue (*n* = 120)
Age, years				
Median (IQR)	74.5 (8.4)	70.8 (8.2)	75.5 (12.8)	73.1 (9.9)
Range	65.1–82.3	56.2–85.9	65.3–84.5	59.9–90.1
Education, years				
Median (IQR)	16.0 (3.5)	16.0 (4.0)	18.0 (1.5)	17.0 (4.0)
Range	12.0–20.0	8.0–20.0	13.0–20.0	12.0–20.0
Frailty index				
Median (IQR)	0.21 (0.12)*	0.18 (0.08)*	0.23 (0.07)*	0.15 (0.13)*
Range	0.08–0.33	0.00–0.41	0.05–0.38	0.03–0.38
mPACC				
Median (IQR)	− 0.32 (2.55)	0.74 (3.56)	0.04 (1.50)	− 0.19 (3.77)
Range	− 7.01–3.46	− 6.39–5.35	− 8.95–3.37	− 7.98–5.16
GDS-15				
Median (IQR)	2.0 (1.0)*	0.0 (1.0)*	1.0 (1.8)	0.0 (1.0)
Range	0.0–5.0	0.0–6.0	0.0–4.0	0.0–5.0
ApoE ε4 carrier status, *N* (%)				
Carriers	11 (36.7)	43 (34.1)	6 (42.9)	28 (23.3)
Non-carriers	19 (63.3)	83 (65.9)	8 (57.1)	92 (76.7)
CSF Aβ_1-42_, pg/ml				
Median (IQR)	1142.0 (872.2)	1239.0 (847.9)	1435.0 (944.1)	1352.0 (746.4)
Range	416.8–1700.0	203.0–1700.0	301.5–1700.0	318.3–1700.0
CSF T-tau, pg/ml				
Median (IQR)	243.6 (107.5)	212.4 (139.3)	213.9 (58.1)	210.1 (100.1)
Range	88.7–501.3	106.7–590.1	131.2–374.9	81.5–492.1
CSF ^181^P-tau, pg/ml				
Median (IQR)	21.9 (12.2)	19.2 (13.3)	20.7 (7.1)	19.3 (9.6)
Range	8.5–53.3	9.6–60.0	10.7–33.3	8.0–53.4
MRI hippocampus volume				
Median (IQR)	4.8 (0.8)*	5.3 (0.8)*	4.8 (0.7)	4.9 (0.7)
Range	3.2–5.9	3.2 (6.5)	4.3 (5.7)	3.6 (6.5)
MRI WMH volume				
Median (IQR)	0.3 (0.4)	0.2 (0.3)	0.2 (0.2)	0.2 (0.3)
Range	0.1–3.7	0.1–2.5	0.1–4.4	0.1–6.9
^18^FDG PET, metaROI				
Median (IQR)	1.3 (0.1)	1.3 (0.1)	1.3 (0.1)	1.3 (0.1)
Range	1.1–1.7	1.1–1.6	1.1–1.5	1.0–1.6
^18^F-AV-45 PET, SUVR				
Median (IQR)	1.1 (0.3)	1.1 (0.2)	1.0 (0.1)	1.0 (0.1)
Range	0.9–1.6	0.9–2.0	1.0–1.5	0.9–1.6

*Aβ*_*1-42*_, amyloid beta_1-42_; *ApoE*, apolipoprotein E; *CSF*, cerebrospinal fluid; ^*18*^*F-AV-45*, florbetapir; ^*18*^*FDG*, ^18^fluorodeoxyglucose; *GDS*, Geriatric Depression Scale; *IQR*, interquartile range; *mPACC*, modified Preclinical Alzheimer’s Cognitive Composite; *MRI*, magnetic resonance imaging; *PET*, positron emission tomography; ^*181*^*P-tau*, ^181^phospho-tau; *ROI*, region of interest; *SUVR*, standardized uptake value ratio; *T-tau*, total tau; *WMH*, white matter hyperintensities

^*^p < 0.05 at the Mann–Whitney test

**Fig. 1:**
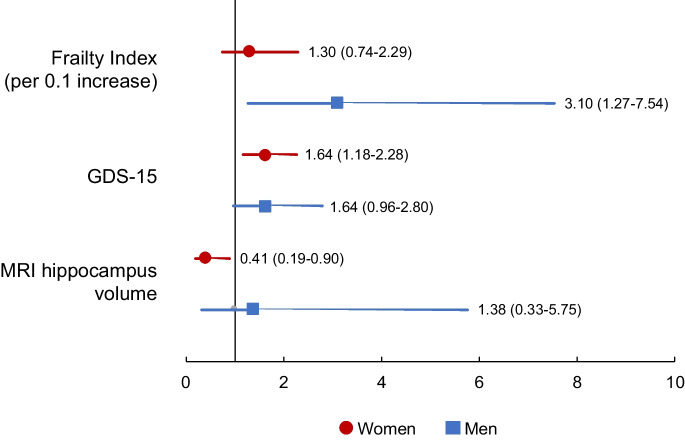
Logistic regression model exploring the factors associated with fatigue (dichotomized dependent variable of interest) by sex. Data are shown as odds ratios (95% confidence intervals). GDS, Geriatric Depression Scale; MRI, magnetic resonance imaging

In a logistic regression model stratified by sex and adjusted by age and education, the variables that resulted significantly associated with fatigue (dichotomized dependent variable of interest) in women were higher GDS-15 scores (OR 1.64, 95% CI 1.18–2.28; *p* < 0.01) and lower MRI hippocampus volumes (OR 0.41, 95% CI 0.18–0.90; *p* = 0.03). Among men, fatigue was significantly associated with higher FI scores (OR 3.10, 95% CI 1.27–7.34 per 0.1 increase; *p* < 0.01). A trend toward statistical significance was also found in the association between fatigue and GDS-15 scores (OR 1.64, 95% CI 0.96–2.80; *p* = 0.07) (Table 2 and Fig. 1).

**Table 2:** Logistic regression model exploring the factors associated with fatigue (dichotomized dependent variable of interest) stratified by sex

	Women (*n* = 30)			Men (*n* = 14)		
	OR	95% CI	*p*	OR	95% CI	*p*
Age	1.02	0.94–1.11	0.68	1.05	0.94–1.18	0.37
Education	0.93	0.79–1.10	0.41	1.23	0.90–1.68	0.20
Frailty index (per 0.1 increase)	1.30	0.74–2.29	0.36	3.10	1.27–7.54	0.01
GDS-15	1.64	1.18–2.28	< 0.01	1.64	0.96–2.80	0.07
MRI hippocampus volume	0.41	0.19–0.90	0.03	1.38	0.33–5.57	0.66

*GDS*, Geriatric Depression Scale; *MRI*, magnetic resonance imaging

## Discussion

The present study aimed to comprehensively investigate the clinical and neurobiological correlates of fatigue in cognitively unimpaired older adults. Overall, our findings align with previous studies indicating fatigue as a complex symptom associated with multiple determinants, encompassing mood, frailty, and neurodegenerative changes. A multidimensional approach is therefore required to achieve a better understanding and management of this highly prevalent manifestation. Noticeably, significant sex differences were found in fatigue prevalence and associated variables suggesting that different pathophysiological mechanisms may underlie fatigue in the two sexes. A sex-informed approach is thus needed to achieve a better understanding of this symptom and develop personalized management strategies.

In line with most of the available evidence, women were more likely to experience/report fatigue than men [11, 52]. Such a higher frequency of fatigue in women has already been attributed to biological factors (e.g., postmenopausal imbalance/decline of sex hormones) [53] and gender-related factors (e.g., educational level, socioeconomic position, household responsibilities). The lower prevalence of self-reported fatigue in men might also be influenced by an already documented sex-driven reporting bias in surveys, with women more willing to participate and more frequently reporting bodily distress and somatic as well as depressive symptoms than men [54, 55]. Of interest, these differences in reporting symptoms exist independently by the time period asked about, the question format/tool used, and the type of collection (e.g., retrospective vs prospective) [54].

An association between fatigue and depression was observed in both sexes (although it did not reach statistical significance in men). Fatigue is identified among the presenting symptoms of major depressive disorders [56]. In turn, fatigue may fuel depression in a bidirectional relationship. Dysfunction in serotoninergic and dopaminergic circuits, the hypothalamic–pituitary–adrenal axis, and the inflammatory cascades may be involved in shared neuropathophysiological pathways. However, fatigue is scarcely responsive to antidepressant treatment, it may emerge as an undesirable effect of psychotropic drugs or polypharmacy and frequently persists as a residual symptom even after depression is resolved. Thus, separate etiologies may also trigger and underlie these phenomena.

In women, fatigue was associated with lower hippocampal volumes on MRI. This finding is consistent with a pilot cross-sectional study showing that fatigue predicts cortical temporal thickness reduction in cognitively normal middle-aged and older adults [57]. Accordingly, fatigue has been recently proposed as an indicator of accelerated brain aging [27] and could represent one of the first manifestations of an incoming neurodegenerative process. The non-significant association with ApoE genotype and biomarkers of amyloid and tau pathology suggests that fatigue may not be specific to AD-related neuronal loss. However, previous studies reported an association of fatigue with ApoE ε4 carrier status and brain amyloid load [25, 26]. Furthermore, the former observation of a relationship between fatigue and white matter lesion volume was not replicated in the present analyses [58].

In men, fatigue was found to be associated with higher frailty levels. Fatigue stands out as one of the core self-reported changes within aging, is a common manifestation of various age-related diseases, and is frequently used as a defining criterion in widely adopted operationalizations of frailty [19]. Fatigue might represent one of the first signs of the age-related disruption of homeostatic reserves and the consequent failure to cope with stressors. As such, it may precede other more clinically evident and measurable manifestations of functional loss (e.g., cognitive impairment, falls, gait disorders, weight loss). In this context, fatigue could serve as a marker of acceleration in aging, potentially aiding in the identification of frail people at a higher risk of poorer outcomes. Furthermore, the impact of this male-specific correlation with frailty might be even greater than observed due to the above-mentioned higher degree of underestimation of somatic complaints among men [52]. Accordingly in a multidimensional assessment of male health, more effort should be focused on actively looking for fatigue in men in order to identify those more vulnerable and frailer.

Based on our findings on sex-specific mechanisms behind the clinical manifestation of fatigue, it is time to envision a solution to tackle and diversify the assessment and management of this symptom in men and women. They imply the need to develop sex-specific care pathways to address fatigue in older persons. Nevertheless, this opportunity requires ad hoc studies to further understand how a public health action against fatigue might adequately balance its subjective nature, the heterogeneity of available instruments, the lack of clear evidence-based interventions, and the sex-specific pathophysiological mechanisms.

Several limitations of the present study should be acknowledged and discussed. Fatigue was ascertained using a single, self-reported question. In this regard, more structured assessment tools have been developed to identify fatigue with higher accuracy. In addition, the adopted definition of fatigue does not consent to fully distinguish fatigue from related constructs (e.g., energy, fatigability). However, given its subjective nature, self-reporting remains an appropriate way of evaluation. The limited sample size (particularly the low number of participants with fatigue) threatens the validity and generalizability of the findings. In addition, the study considered a population of relatively healthy and highly educated older adults who are poorly representative of the individuals encountered in daily clinical practice. However, ADNI offered the unique opportunity to explore our research questions in a population well characterized clinically and biologically. The cross-sectional design impedes determining any causal relationship between fatigue and the associated variables. Future longitudinal studies are thus needed to confirm and expand our findings. Moreover, the observed results might be influenced by a sex-driven reporting bias that might underestimate the impact of conditions such as fatigue and depression among men [59]. Finally, we did not consider gendered sociocultural variables possibly contributing to the observed sex differences [60].

The main study strength instead lies in the broad exploration of fatigue’s multidimensionality, resulting in analyzing a wide array of potential correlates. This approach has allowed us to grasp how fatigue probably results from the presence and interaction of multiple clinical and subclinical underpinnings and cannot be fully explained by standalone determinants. Ideally, this comprehensive framework should be translated into clinical practice where the screening of fatigue could enable the early identification of at-risk individuals and the detection of pathological conditions that could benefit from timely interventions. However, it appears crucial to determine when fatigue assumes negative clinical and prognostic implications to avoid overdiagnosis.

## Conclusion

Fatigue is a complex symptom, probably resulting from the interplay between diverse determinants including depression, frailty, and brain changes. Moreover, it seems to be differently expressed in the two sexes. The multifaceted nature of fatigue underscores the need for a comprehensive and multidimensional approach to explore its underlying mechanisms and broader implications. Specifically, a sex-informed perspective seems necessary in the premise of personalized management.

## Supplementary Information

Below is the link to the electronic supplementary material.

Supplementary file1 (PDF 123 KB)

## Data Availability

The data supporting the findings of this study are available on request from the corresponding author. The data are not publicly available due to privacy or ethical restrictions.

## Abbreviations

AD: Alzheimer’s disease
ADNI: Alzheimer’s Disease Neuroimaging Initiative
ApoE: Apolipoprotein E
CSF: Cerebrospinal fluid
FI: Frailty Index
GDS: Geriatric Depression Scale
MCI: Mild Cognitive Impairment
MMSE: Mini-Mental State Examination
MRI: Magnetic Resonance Imaging
PET: Positron Emission Tomography
