# Supplementary material for: Sex-specific clinical and neurobiological correlates of fatigue in older adults
Source: GeroScience. 2024 Aug 12;47(1):1151–60. doi: 10.1007/s11357-024-01259-0 (PMC11872861; doi:10.1007/s11357-024-01259-0)
Supplement: Supplementary file 1 — Supplementary file1 (PDF 123 KB) [file 11357_2024_1259_MOESM1_ESM.pdf]

Sex-specific clinical and neurobiological correlates of fatigue in older adults

Marco Toccaceli Blasi[1°], Alba Rosa Alfano[2°], Martina Salzillo[1], Simona Buscarnera[1], Valeria Raparelli[3], Matteo Cesari[4], Giuseppe Bruno[1,5], Marco Canevelli[1,6], for the Alzheimer's Disease Neuroimaging Initiative*

Affiliations

[1] Department of Human Neuroscience, "Sapienza" University, Rome, Italy

[2] Department of Internal Medicine and Medical Specialties, UOC Geriatrics, Sapienza University, Rome, Italy

[3] Department of Translational Medicine, University of Ferrara, Ferrara Italy; Department of Translational and Precision Medicine, Sapienza University of Rome, Rome, Italy

[4] Ageing and Health Unit; Department of Maternal, Newborn, Child, Adolescent Health and Ageing; World Health Organization

[5] Santa Lucia Foundation IRCCS, Rome, Italy.

[6] National Center for Disease Prevention and Health Promotion, Italian National Institute of Health, Rome, Italy; Aging Research Center, Department of Neurobiology, Care Sciences and Society, Karolinska Institutet and Stockholm University, Stockholm, Sweden

°These Authors equally contributed to the paper

*Data used in the preparation of this article were obtained from the Alzheimer's Disease Neuroimaging Initiative (ADNI) database (adni.loni.usc.edu). As such, the investigators within the ADNI contributed to the design and implementation of ADNI and/or provided data but did not participate in the analysis or writing of this report. A complete listing of ADNI investigators can be found at: http://adni.loni.usc.edu/wp-content/uploads/how_to_apply/ADNI_Acknowledgement_List.pdf

Corresponding author

Marco Toccaceli Blasi, MD

Department of Human Neuroscience, "Sapienza" University, Rome, Italy

Viale dell'Università 30, 00185 Rome, Italy

E-mail: marco.toccaceliblasi@uniroma1.it

Tel: +39 3394727161

Supplementary material

Table S1. Deficits considered in the computation of the Frailty Index

Items	Scoring 0	0.25	0.5	1
1. Renal-Genitourinary diseases	No	-	-	Yes
2. Dermatologic-Connective diseases	No	-	-	Yes
3. Hepatic diseases	No	-	-	Yes
4. Cardiovascular diseases	No	-	-	Yes
5. Endocrine-Metabolic diseases	No	-	-	Yes
6. Neurological (non-AD) diseases	No	-	-	Yes
7. Psychiatric diseases	No	-	-	Yes
8. Malignancies	No	-	-	Yes
9. Musculoskeletal diseases	No	-	-	Yes
10. Gastrointestinal diseases	No	-	-	Yes
11. Respiratory diseases	No	-	-	Yes
12. Head, Eyes, Ears, Nose and Throat diseases	No	-	-	Yes
13. Hematopoietic-Lymphatic diseases	No	-	-	Yes
14. Urinary discomfort	Absent	-	-	Present
15. Shortness of breath	Absent	-	-	Present
16. Falls	Absent	-	-	Present
17. Insomnia	Absent	-	-	Present
18. Constipation	Absent	-	-	Present
19. Drowsiness	Absent	-	-	Present
20. Dizziness	Absent	-	-	Present
21. Musculoskeletal pain	Absent	-	-	Present
22. Seated BP Diastolic	≤90mmHg	-	-	>90mmHg
23. Seated BP Systolic	≤140mmHg	-	-	>140mmHg
24. Tremor	Absent	-	-	Present
25. Motor Strength	Normal	-	-	Abnormal
26. Gait	Normal	-	-	Abnormal
27. Cerebellar - Finger to Nose	Normal	-	-	Abnormal
28. Agitation/Aggression (NPI)	No	-	-	Yes
29. Anxiety (NPI)	No	-	-	Yes
30. Apathy/Indifference (NPI)	No	-	-	Yes
31. Irritability/Lability (NPI)	No	-	-	Yes
32. Heating water, making a cup of coffee (FAQ)	0	1	2	3
33. Traveling out of the neighborhood (FAQ)	0	1	2	3
34. Preparing a balanced meal (FAQ)	0	1	2	3
35. Writing checks, paying bills, or balancing checkbook (FAQ)	0	1	2	3
36. Paying attention to and understanding a TV program, book, or magazine (FAQ)	0	1	2	3
37. Playing a game of skill such as bridge or chess (FAQ)	0	1	2	3
38. Shopping alone for clothes, household (FAQ)	0	1	2	3
39. Assembling tax records, business affairs (FAQ)	0	1	2	3

AD, Alzheimer Disease; FAQ, Functional Activities Questionnaire; NPI, Neuropsychiatric Inventory.
